# Inter-call intervals, but not call durations, adhere to Menzerath’s Law in the submissive vocal bouts of meerkats

**DOI:** 10.1098/rsos.241351

**Published:** 2024-12-11

**Authors:** Stuart Kyle Watson, Mara Zali, Nikola Falk, Paul Widmer, Marta B Manser

**Affiliations:** ^1^ Department of Evolutionary Biology and Environmental Studies, University of Zürich, Winterthurerstrasse 190, Zurich 8057, Switzerland; ^2^ Center for the Interdisciplinary Study of Language Evolution, University of Zürich, Zürich, Switzerland; ^3^ Department of Comparative Language Science, University of Zürich, Affolternstrasse 56, Zürich 8050, Switzerland; ^4^ Kalahari Research Centre, Kuruman River Reserve, Northern Cape, South Africa; ^5^ Mammal Research Institute, University of Pretoria, Hatfield, Pretoria 0002, South Africa

**Keywords:** animal communication, vocal behaviour, Menzerath’s Law, quantitative linguistics

## Abstract

Diverse information encoding systems, including human language, the vocal and gestural systems of non-human animals and the structure of DNA and proteins, have been found to conform to ‘Menzerath’s Law’—a negative relationship between the number of units composing a sequence, and the size of those units. Here, we test for the presence of Menzerath’s Law in the vocal bouts produced in a submissive context by meerkats (*Suricata suricatta*). Using a suite of Bayesian mixed effects models, we examined 1676 vocal bouts produced by 89 wild meerkats, ranging from 1 to 590 calls in length, to determine whether the number of calls composing each bout had a negative relationship with the duration of those calls or their inter-call intervals. In contradiction to Menzerath’s Law, we found that the duration of vocalizations had a positive relationship with the number of calls in a bout. However, the duration of intervals between calls did have a negative relationship with bout size. Moreover, both calls and intervals had longer durations the closer they were positioned to the end of the bout. These findings highlight the multi-faceted ways in which efficiency trade-offs can occur in the vocal repertoires of non-human animals, shaping variability in the production of signal forms.

## 1. Introduction

A curious statistical phenomenon known as ‘Menzerath’s Law’ has been found to hold across a disparate range of naturally occurring information-coding systems, which states that as the relative size of a combinatorial construct increases, the size of its composing units will decrease. Having long been established to hold true in the vast majority of human languages [1,2], the scope of Menzerath’s Law has since expanded to include music [3], vocal and gestural communication in animals [4] and even biological structures such as genes [5] and proteins [6,7]. The ubiquity of this statistical relationship suggests that there is a universal selection pressure for efficiency in information-coding systems (although exceptions to the rule also exist [4,8]). Exploring the prevalence of Menzerath’s Law across a broad range of communication systems, identifying adherence and exceptions, and the factors likely to be driving these patterns, will provide valuable insights into the selective pressures that shape the evolution of communicative behaviour [4,9].

Previous work has found evidence for Menzerath’s Law in the vocal and gestural repertoires of diverse species including primates [10–16], African penguins (*Spheniscus demersus*) [17], songbirds [18] and bottlenose dolphins (*Tursiops truncates*) [19] (see electronic supplementary material, table S1 for summary of previous studies). However, the signals of some species demonstrate exceptions or mixed evidence for Menzerath’s Law. For instance, Menzerath’s Law in the feeding calls of wild mountain gorillas (*Gorilla beringei beringei*) [20] was only statistically extant if bouts of a single call were included for analysis (something typically removed in linguistic analyses), and if not then a weakly positive relationship between sequence size and call duration was identified instead. Meanwhile, Clink & Lau [8] found that, in three species of primate (tarsiers, *Tarsius spectrumgurskyae*; titi monkeys, *Plecturocebus cupreus;* gibbons, *Hylobates funereus*), Menzerath’s Law was only present in a minority (two/eight) of the call types examined. One explanation offered by Clink & Lau for these exceptions is that the call types examined were long-distance calls, which may therefore have adapted to optimize salient transmission of the call rather than for energetic efficiency [8,21,22]. In songbirds, patterns consistent with Menzerath’s Law were identified across 15 species [18], but the authors found these patterns could be explained by a variety of mechanisms not directly related to information compression, highlighting in particular the role that ease of motor production of vocal units is likely to play. This growing body of work demonstrates the importance of exploring Menzerath’s Law in an increasingly diverse range of species and call types. In doing so, we will be able to identify the factors contributing towards the evolution of signal forms both within and across species [21].

**Figure 1.**
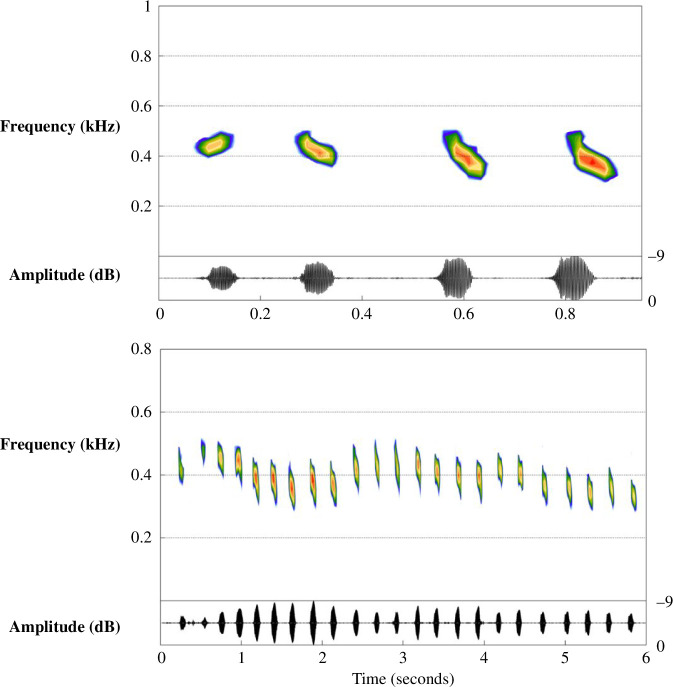
Two spectrogram examples of typical calling bouts produced during a submissive encounter, consisting only of short-note calls. The top bout contains four calls, and the bottom contains 23. Spectrograms of other call types sometimes found in submissive bouts can be found in electronic supplementary material, figure S1. Spectrograms produced using a fast Fourier transform (FFT) size of 1024 points, Hanning window and 50% window overlap in the R package ‘seewave’ [30]. Example recordings of bouts are available at the following OSF repository: https://osf.io/vjgke/.

To expand the taxonomic survey of Menzerath’s Law, we investigate its presence in the vocal behaviour of meerkats (*Suricata suricatta*), a cooperatively breeding species of mongoose. Meerkats have a vocal repertoire of over 30 known call types [23], some of which may be combined to augment or change the function of the signal [24,25]. As a highly social, cooperatively breeding species that can live in large groups of up to 50 individuals [26], vocal behaviour is an important component in group coordination and the navigation of social contexts [23]. The social structure of meerkats can be described as ‘despotic’ [27], with strict dominance hierarchies within each sex, enforced through aggressive behaviour by dominant individuals. Subordinate individuals regularly produce bouts of vocalizations during times of social tension with same-sex dominant individuals (e.g. when approaching them, or being approached by them), often in combination with a submissive body posture and/or grooming or following the receiver [28,29]. This behaviour presumably serves as a means of placating the dominant individual and avoiding or de-escalating an aggressive situation [25,26]. These bouts typically comprise rapid, high-pitched ‘short note’ calls (see figure 1) [23,24,29] but may occasionally also contain other call types including alarm calls, contact calls and movement-related (‘lead’ and ‘move’) calls. Whether these mixtures of call types are ever combinatorial (i.e. the combination changes the function of the signal), or rather instead reflect competing or overlapping internal and/or contextual states for the signaller, is currently unknown. Because these bouts are highly variable in terms of how many calls they contain [24], they make an excellent test case for exploring Menzerath’s Law.

## 2. Methods

### 2.1. Study population

We collected our data at the Kalahari Meerkat Project, Kuruman River Reserve, South Africa (26°58’ S, 21°50’ E) over almost 6 years (November 2013 to March 2019). Audio recordings were collected from 89 female adult (aged 2 years or older) individuals from 16 groups of wild meerkats (ranging from 6 to 24 individuals per group, overall *n* = 89), all of whom were well habituated to the presence of humans. Because female meerkats typically remain in their natal group, the vast majority of callers would be closely related to the target receiver. Only females were followed, as this dataset was originally collected to study intra-sexual reproductive conflict.

### 2.2. Data collection

Data was recorded during group follows using a Sennheiser ME66 directional microphone (40–20 000 Hz) on a telescopic pole, which was connected to a Marantz PMD661 MKII solid-state recorder (sampling frequency 48 kHz, 16−24 bit in .wav format). A ‘submissive event’ was coded as each time a subordinate female entered within 5 m of the dominant female and vocalized. Submissive events ended when the subordinate and dominant were no longer within 5 m of each other, or vocalizations ceased. Because these events were recorded opportunistically from focal individuals, individuals they interacted with, and other dyadic interactions happening around them, we cannot report a representative number of sampling hours per individual. All calls were recorded from a distance of 0.2–5 m from the vocalizing individual. A total of 27 572 individual calls across 1676 vocal bouts were recorded (see §2.3 for details of how bouts were determined) in 529 independent events. See electronic supplementary material, figure S2 for the frequency with which each bout size was sampled. Of the 27 572 calls recorded, 1075 (approx. 4% of the total) were call types other than short notes (move calls [31] = 148, grooming calls [32] = 138, close calls [33] = 73, di-drr calls [34] = 44 and lead calls [31] = 207). Recordings were annotated by M.Z. in Adobe Audition 2015 [35], identifying the call type by ear and examination of spectrograms, and determining the beginning and end of calls by visual assessment of the spectrogram (temporal resolution = 0.001 s, pitch range = 425–1650 Hz). Metadata from these annotations was then batch-extracted for analysis using the software ‘ExifTool’ (version 12.63) [36]. Some of this dataset has been used for analyses in other published work [24].

### 2.3. Statistical analysis

#### 2.3.1. Bout criterion determination

To determine whether two or more calls produced in succession should be considered as belonging to the same bout, or two separate bouts, we used the maximum-likelihood approach described by Langton *et al*. [37] and Luque & Guinet [38], using the R package ‘diveMove’ [39] to perform the calculations. Using this approach, a bout criterion of 1.25 s was determined (see electronic supplementary material, figure S4 for the distribution of intervals in raw data). Therefore, if the inter-call interval between a call and a subsequent call (regardless of whether they were the same call type) in the same recording was equal to or less than 1.25 s, they were coded as being part of the same bout. Accordingly, calls that follow an inter-call interval of longer than 1.25 s were coded as being the first call in a new bout. Each bout was assigned a unique bout identifier code and the number of calls composing each bout was computed. Each of the 89 callers contributed a mean of 310 individual calls (s.d. = 83), and a mean of 18.8 bouts (s.d. = 48.8).

#### 2.3.2. Statistical models

We used Bayesian Markov chain Monte-Carlo (MCMC) generalized linear mixed models (‘GLMMs’, using the package ‘brms’ [40]) to explore the relationship between the duration of each call that comproes each vocal bout (dependent variable), and the number of calls contained in that bout. Because it was highly plausible that different call types may be influenced differently by Menzerath’s Law [8], all models included a parameter (‘call-length category’) for whether each individual call belonged to a ‘short’ (average durations—short notes: 0.038 s, ‘di-drr’ calls: 0.089 s) or ‘long’ call types (average durations—close calls: 0.160 s, grooming calls: 0.201 s, lead calls: 0.177 s and move calls: 0.167 s). We observed no obvious difference between long and short call types in the length of bout they were most often found in (electronic supplementary material, figure S3 shows the proportion of ‘long’ and ‘short’ call types sampled at each bout size). We ran a model where this call-length category parameter was fit with an interaction effect with the number of calls in a bout. The models also contained a parameter encoding the logged position of the call within the bout, to explore whether being positioned closer to the beginning or end of the bout influenced call duration (this parametrization is hereafter referred to as the ‘full model’). These full models were compared with a ‘null’ model, which did not contain a fixed effect of the number of calls within a bout (but were otherwise identical to the full model), to determine which model provided the best out-of-sample predictive fit for the data. All full and null models included random intercepts for caller identity, bout identity and recording identity (table 1). Models were run with two chains of 4000 iterations each. Trace plots, ‘r-hat’ values and effective sample sizes were used to assess model convergence. Priors were chosen to be weakly regularizing to control for both under- and over-fitting the model to the data. For each analysis, the full and null models were compared to determine which had the best out-of-sample predictive fit using the difference in generic (expected) log-predictive density (ELPD) between models. Where there was no clear best-fitting model (i.e. the difference in ELPD was less than twice the standard error of the difference in ELPD between models), the most parsimonious model (i.e. the null model) was adopted for inference.

**Table 1.** Model structures used for call duration analysis. Models used for the inter-call interval analysis described below were identical except for the outcome measure, which was log(duration of inter-call interval).

model	outcome measure	predictors	random intercepts
full model	log(duration of call)	number of calls in bout *	caller ID, bout ID, recording ID
		call-length category,	
		logged position of call within bout	
null	log(duration of call)	call-length category,	caller ID, bout ID, recording ID
		logged position of call within bout	

* indicates an interaction effect.

To control for the possibility that statistical identification of Menzerath’s Law is strongly influenced by the presence of specific data points [20], we ran four analyses using different configurations of the overall dataset. Specifically, we ran analyses using:

1. All recorded bouts (*n* = 1676 bouts).
2. All bouts containing more than a single call, as utterances of a single word, are often excluded from linguistic studies of Menzerath’s Law [2,4,9] (*n* = 1380 bouts).
3. Bouts with a number of calls up to the point where 90% of the data is accounted for, to explore Menzerath’s Law in the absence of potentially influential outliers in bout length (max bout length = 35 calls, *n* = 1509 bouts).
4. Bouts with a number of calls up to the point where 90% of the data is accounted for, after all bouts of a single call had been removed (max bout length = 42 calls, *n* = 1243 bouts).

For some recordings, it is possible that the observer was late in recognizing the start of a submissive bout, resulting in a slight underestimation of the total number of calls for the first bout in that recording. Therefore, to be conservative, we reran our analysis excluding the first bout in each recording, leaving a total of 1147 bouts and 14 054 calls, but this did not change our overall results, except that the interaction effect was less stable across datasets (see electronic supplementary material, tables S2 and S3 for outputs).

Additionally, we wished to explore the possibility that compression resulting from Menzerath’s Law might act at the level of inter-call intervals rather than call durations in meerkats, as has been observed in geladas [12]. To do this, we reran the analysis described above, using log-transformed inter-call intervals as a response measure, while keeping all other aspects of the analysis identical. Because analysis of inter-call intervals necessarily entails examining bouts of more than a single call, this was carried out with subsets of the full dataset containing (i) all bouts with more than 1 call and (ii) all bouts with more than 1 and less than 42 calls (90% of the data). In this analysis, the parameter for long versus short call-length categories refers to the call immediately preceded by the inter-call interval.

All raw data and R scripts used for analysis can be downloaded from our Open Science Framework repository:https://osf.io/vjgke/.

## 3. Results

**Figure 2.**
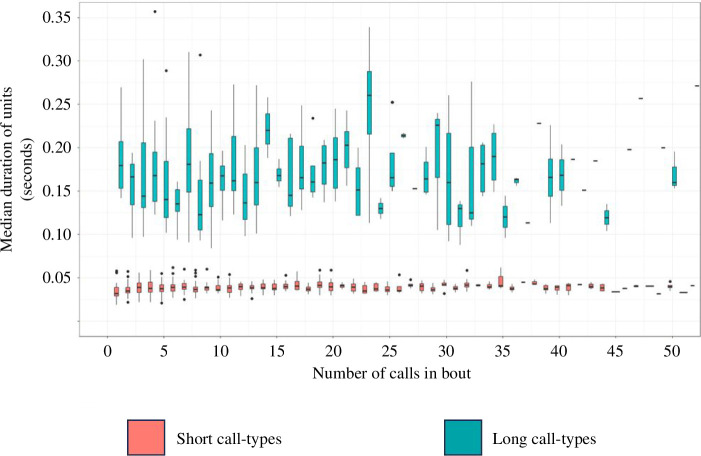
Median duration of calls produced according to the number of calls contained in the bout. Only bouts of less than 50 calls (accounting for approx. 95% of observations) are included to improve readability. Very narrow boxes are likely to indicate a small sample for the corresponding x-axis value. See electronic supplementary material, figure S5 for a plot of the full dataset.

We analysed 1676 vocal bouts containing between 1 and 590 calls (median = 5 calls, see electronic supplementary material, figure S2 for sampling distribution). In total, 90% of bouts had 35 or fewer individual vocalizations within them. Across all bouts, calls had a median duration of 0.038 s (minimum: 0.012 s, maximum: 0.468 s, figure 2). Collectively, short call types had an average duration of 0.37 s (minimum: 0.012 s, maximum: 0.064 s) and long call types had an average duration of 0.164 s (minimum: 0.084 s, maximum: 0.468 s).

**Figure 3.**
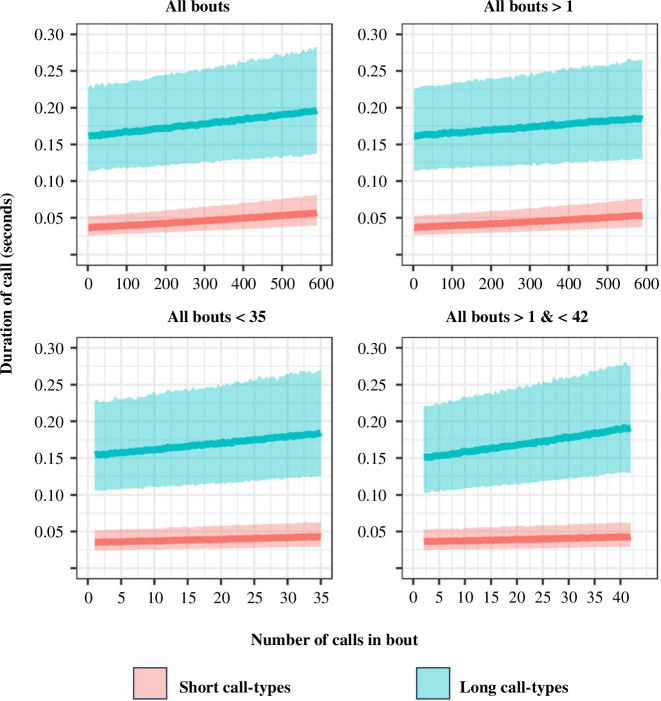
Posterior predictive plots for all four full models from tables 2 and 3. Outcome measure back-transformed from the log scale used for analysis to aid interpretation.

Regardless of whether examining the full or truncated datasets for analysis, the full models provided superior out-of-sample predictive fit relative to the null model in all cases (see table 2). In each full model, the average duration of calls for both short and long categories had a positive relationship with the number of calls in a bout (table 3, figure 3). There was also an interaction effect of call-length category and number of calls in bout, indicating the effect of bout size was greater for ‘long’ category calls (figure 3). This evidence of a positive relationship between the number of calls in a vocal bout and the median duration of calls composing it directly contradicts the predictions of Menzerath’s Law. Finally, the position of the call within its bout was also found to have a positive effect, with calls closer to the end of the bout typically having a greater duration than those with an earlier position.

**Table 2.** Table showing model comparison outputs for each set of models for each subset of the dataset, in descending order of out-of-sample predictive power as determined by ELPD. A difference in ELPD of greater than two times the standard error of the difference in ELPD would suggest the model provides a substantially better predictive fit than the competing model. In the absence of this, the null model is adopted as the most parsimonious. Coefficients for best-fitting models can be found in table 3.

data	model	ELPD difference from best model	s.e. difference from best model
all data (*n* = 1678)	full	–	–
	null	−35.36	13.12
only bouts > 1 call (*n* = 1380)	full	–	–
	null	−27.49	11.89
bouts < 35 calls (*n* = 1509)	full	–	–
	null	−33.53	7.64
bouts > 1 and < 42 calls (*n* = 1243)	full	–	–
	null	−24.28	7.47

**Table 3.** Outputs for full model estimates (table 2). A robust effect can be ascertained according to whether the 95% credible intervals cross zero for a given parameter. Values are given to four decimal places rather than standard three because values were very small, but reducing to e.g. ‘<0.001’ would conceal important detail. Posterior predictive plots based on outputs are shown in figure 3.

**data**	**parameter**	**estimate**	**estimate error**	**lower 95% credible interval**	**upper 95% credible interval**
all data (*n* = 1678)	intercept	−3.2841	0.014	−3.3116	−3.2563
	number of calls in bout	0.0007	0.0001	0.0006	0.0009
	call-length category (long/short)	1.4814	0.0079	1.4658	1.4969
	logged position of call in bout	0.0222	0.0012	0.0199	0.0247
	number of calls in bout * call-length category	−0.0004	0.0001	−0.0005	−0.0003
bouts > 1 (*n* = 1380)	intercept	−3.2738	0.014	−3.3008	−3.2461
	number of calls in bout	0.0006	0.0001	0.0005	0.0008
	call-length category (long/short)	1.4734	0.0082	1.4575	1.4896
	logged position of call in bout	0.0229	0.0012	0.0205	0.0253
	number of calls in bout * call-length category	−0.0004	0.0001	−0.0005	−0.0003
bouts < 35 (*n* = 1509)	intercept	−3.3182	0.0155	−3.3488	−3.2876
	number of calls in bout	0.0058	0.0005	0.0048	0.0067
	call-length category (long/short)	1.4739	0.0176	1.44	1.508
	logged position of call in bout	0.0389	0.0025	0.0341	0.0438
	number of calls in bout * call-length category	−0.0005	0.001	−0.0025	0.0014
bouts > 1 and < 42 (*n* = 1243)	intercept	−3.2981	0.0155	−3.328	−3.2683
	number of calls in bout	0.0042	0.0004	0.0035	0.005
	call-length category (long/short)	1.4248	0.0176	1.39	1.4587
	logged position of call in bout	0.038	0.0023	0.0334	0.0425
	number of calls in bout * call-length category	0.0017	0.0008	0.0002	0.0032

* indicates an interaction effect.

**Figure 4.**
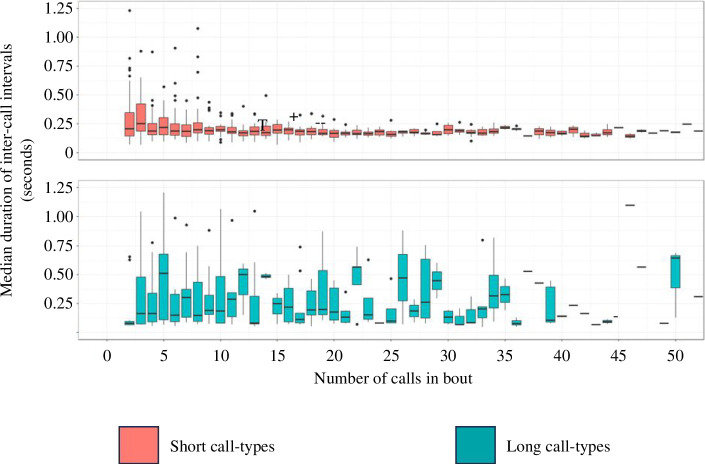
The median duration of inter-call intervals depending on the total number of intervals existing within a vocal bout. Only bouts of less than 50 calls (accounting for approx. 95% of observations) are included to improve readability. Very narrow boxes are likely to indicate a small sample for the corresponding x-axis value. See electronic supplementary material, figure S6 for full sample.

**Figure 5.**
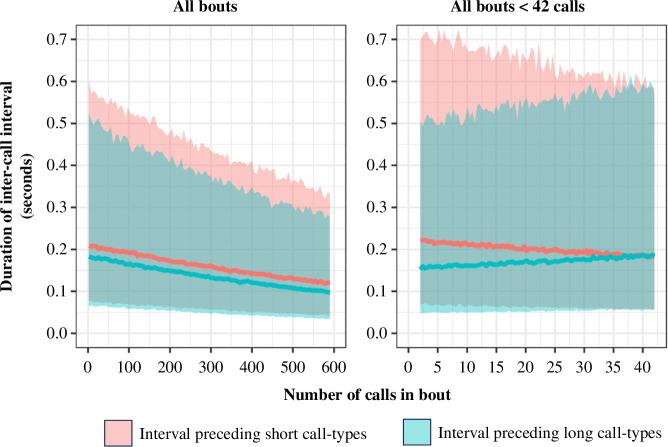
Posterior predictive plots for full models exploring the relationship between the duration of inter-call intervals and bout size (table 5). Outcome measure back-transformed from the log scale used for analysis to aid interpretation.

We also explored whether the meerkats demonstrated compression in the intervals of silence between calls within a bout. Across all bouts, inter-call intervals preceding short-category calls (*n* = 24 991) had a median duration of 0.173 s (minimum = 0.042 s, maximum = 1.249 s), while intervals preceding long-category calls (*n* = 905) had a median duration of 0.155 s (minimum = 0.039 s, maximum = 1.243 s, figure 4). In this analysis, the full model provided the best out-of-sample predictive fit for the data regardless of which dataset was used (table 4). The full model for the full dataset indicated that inter-call intervals preceding both short and long call types demonstrated a negative relationship with number of calls in a bout (figure 5, table 5), with no interaction effect between number of calls and call-length category. In this model, the relationship between inter-call interval duration and bout size is therefore consistent with Menzerath’s Law for both call-length categories. However, the full model using a dataset that excluded unusually long sequences (i.e. only bouts of less than 42 calls, accounting for 90% of the data) found an interaction between the call-length category following the interval and bout size. Here, intervals preceding short-category calls had a negative relationship with bout size, while intervals preceding long-category calls had a positive relationship. Intervals preceding short call types were therefore consistent with Menzerath’s Law in both datasets, whereas intervals preceding long call types were only consistent with Menzerath’s Law when including large (greater than 42, max 590 calls) bout sizes. Finally, we also found in both full models that the position of the call within its bout had a positive effect on inter-call interval duration, with intervals closer to the end of the bout typically having a greater duration than those in an earlier position (table 5).

**Table 4.** Table showing model comparison outputs for each set of models from analysis of inter-call intervals in descending order of out-of-sample predictive power as determined by ELPD. A difference in ELPD of greater than two times the standard error of the difference in ELPD would suggest the model provides a substantially better predictive fit than the competing model. In the absence of this, the null model would be adopted as the most parsimonious. Coefficients for best-fitting models can be found in table 5.

data	model	ELPD difference from the best model	s.e. of ELPD difference from the best model
only bouts > 1 call (*n* = 1380)	full model	–	–
	null model	−8.14	2.88
bouts > 1 and < 42 calls (*n* = 1243)	full model	–	–
	null model	−14.15	5.82

**Table 5.** Outputs for full model estimates of inter-call interval analysis (table 4). A robust effect can be ascertained according to whether the 95% credible intervals cross zero for a given parameter. Values are given to four decimal places rather than standard three because values were very small, but reducing to e.g. ‘<0.001’ would conceal important detail. Posterior predictive plots based on outputs are shown in figure 5. Asterisk indicates an interaction effect.

**data**	**parameter**	**estimate**	**estimate error**	**lower 95%**	**upper 95%**
				**credible interval**	credible interval
only bouts > 1 call (*n* = 1380)	intercept	−1.5181	0.0202	−1.5585	−1.4796
	number of calls in bout	−0.001	0.0002	−0.0013	−0.0007
	call-length category preceding interval (long/short)	−0.1345	0.0258	−0.186	−0.0851
	logged position of interval in bout	0.0508	0.0042	0.0424	0.0592
	number of calls in bout * call-length category	−0.0001	0.0002	−0.0004	0.0002
bouts > 1 and < 42 calls (*n* = 1243)	intercept	−1.436	0.0237	−1.4828	−1.3904
	number of calls in bout	−0.0047	0.0009	−0.0063	−0.003
	call-length category preceding interval (long/short)	−0.3601	0.0608	−0.4779	−0.2405
	logged position of interval in bout	0.0968	0.0094	0.0788	0.1151
	number of calls in bout * call-length category	0.009	0.0026	0.004	0.0141

* indicates an interaction effect.

## 4. Discussion

We tested for the presence of Menzerath’s Law in the call sequences of wild meerkats emitted in a submission context and, on the contrary, identified a positive relationship between the number of calls within a vocal bout and the duration of those calls. However, we did find evidence of compression at the level of inter-call intervals: Larger bouts typically had shorter intervals when they preceded short call types (single-notes and ‘di-drr’ calls), whereas evidence was mixed for longer call types (lead, grooming, contact and moving calls) depending on whether the entire dataset was examined (bouts of up to 590 calls), or only bouts of the most commonly occurring sizes (less than 42 calls, accounting for 90% of the data). It is worth restating that these longer call types accounted for only 4% of the overall dataset, and so we conclude that Menzerath’s Law predicts the vast majority of inter-call interval durations within submissive calling bouts. Furthermore, we found that both calls and intervals were typically of greater duration the closer they were to the end of the bout. Taken together, these data suggest meerkats may flexibly alter the duration of inter-call intervals to offset longer calls produced in larger vocal bouts.

The finding that meerkat vocalizations did not adhere to Menzerath’s Law adds to a growing body of literature demonstrating exceptions to Menzerath’s Law in the vocal units of non-human species [8,20]. In previous work, Watson *et al*. [20] found that the short-distance feeding calls of gorillas were not subject to Menzerath’s Law, and speculated that this may be due to ‘redundancy’ in the signal allowing them to be interpretable in densely vegetated habitat. Similarly, Clink & Lau [8] argued that the exceptions to Menzerath’s Law identified in the duet songs of various primate species may be explained by their focus on long-distance contact calls whose form is likely to have been optimized for salient transmission rather than efficiency. These explanations are unlikely to be the case in the present study, as the call sequences studied here were produced in very close proximity to the receiver (a dominant conspecific) without dense physical obstructions, and hence are likely to be readily audible. Moreover, a number of long-distance calls in other species have been found to adhere to Menzerath’s Law (e.g. chimpanzee pant-hoots [13], birdsong [18,41] and whalesong [42]; see electronic supplementary material, table S1), suggesting that this factor has not consistently acted as a constraint upon adherence to Menzerath’s Law across species.

One possible explanation for our finding is that calls produced by meerkats during submission contexts are already highly optimized in terms of efficiency (many calls were as short as 0.02 s) due to the emotional urgency of the situation. This optimization may be at the production level, reaching the limits of what can be produced by the vocal apparatus of a meerkat [43], or instead at the receiver level as calls need to be of sufficient duration that receivers can detect them and determine their function by acoustically distinguishing them from other call categories. So-called ‘performance constraints’ [43,44] on the trade-off between call rate and frequency bandwidth have been primarily examined within the context of mating displays, but it is plausible that such trade-offs also occur in contexts such as submission (albeit under weaker selective pressure). Why the calls in our dataset became longer in larger sequences (the opposite relationship to that predicted by Menzerath’s Law) is challenging to explain but might plausibly be a result of heightened emotional states underlying long bouts of vocal behaviour.

Despite a lack of evidence for compression in the duration of meerkat vocalizations, we did find that the intervals of silence between calls adhered to the predictions of Menzerath’s Law. While most studies of Menzerath’s Law have not examined this dimension of vocal behaviour, the intervals in close-ranged affiliative contact call sequences produced by geladas [12] also follow this pattern (although, unlike meerkats, so do the calls themselves). This finding provides additional nuance to our understanding of the flexibility underlying meerkat vocal production, as they are already known to alter call production rate as a response to environmental (e.g. drought [45]) and social factors (e.g. presence of pups [46] and proximity to social partners [47]). Closely related dwarf mongooses (*Helogale parvula*) also increase the rate at which they produce recruitment calls according to contextual urgency [48]. For meerkats, it is therefore plausible that the production of very large bouts in submissive contexts is reflective of heightened arousal states, or that arousal becomes heightened as bouts continue due to the ongoing threat of a proximate dominant individual. One possibility is that the shortening of inter-call intervals in longer bouts compensates for the relatively longer duration of calls produced in these bouts in terms of communicative efficiency. Fine-grained examination of the details of these social interactions, and how they relate to changes in calling behaviour, is likely to further illuminate the motivational factors underlying compression of inter-call intervals. Recent work with common marmosets (*Callithrix jacchus*) has used innovative experimental methods to directly probe the factors underlying another phenomenon known as ‘Zipf’s Law’, whereby the most commonly used elements in a repertoire tend to be the shortest [49]. In this work, the marmosets were trained to produce vocalizations upon a visual cue and it was found that, as training progressed, they significantly increased their rate of calling and deployed shorter call types more frequently, to increase the efficiency of their calling behaviour. Similar methods have yet to be applied to the exploration of Menzerath’s Law but may prove a powerful means of systematically exploring the factors underlying its expression in a controlled fashion.

An intriguing additional detail of our results is that the durations of both calls and inter-call intervals were, on average, longer the closer they were located to the end of the bout. A parallel with this can be found in the speech and music of humans, as well as in birdsong, in a phenomenon known as ‘final lengthening’ [50–52]. In this, units (i.e. vowels and phrases) become progressively lengthened at prosodic boundaries, from non-final, to penultimate, to final. It has been argued that this phenomenon may result from a general property of motor production whereby actions decelerate towards the end [50], which our findings would seem to support. However, exploration of a broader range of systems and behavioural domains would be necessary to draw firm conclusions.

An interesting pattern emerging in the data on Menzerath’s Law in animal vocalizations is that, so far, systems in which sequences are composed primarily of repetitions of a single call type (as is the case in the present study, where only 4% of calls were not short notes) do not adhere to Menzerath’s Law [15]. Conversely, when examining sequences that often or typically comprise more than one call or gesture type, adherence to Menzerath’s Law is widespread across diverse signal types including song (i.e. birds [17,18,41], whales [42]), contact calls (dolphins [19], chimpanzees [13], geladas [12]) and even gestures (chimpanzee play [10] and some sexual solicitation [11] gestures). However, an exception to this comes from data on the heterogeneous food-call sequences of mountain gorillas [20], which did not show strong evidence of Menzerath’s Law. Interestingly, Hainan tree frogs do demonstrate Menzerath’s Law in their multi-call sequences, but only one of the call types composing those sequences is affected by it [53]. Exploration of further vocal systems comprising both homogeneous and heterogeneous call sequences is, therefore, required to determine whether and how this factor plays a role in adherence to Menzerath’s Law. However, one possibility is that the single call-type sequences tested so far can be best understood as a stream of independently motivated vocalizations in close temporal proximity (e.g. an ‘emotional readout’), whereas multiple-call-type sequences are constrained by the way calls must be juxtapositioned in order to be intelligible to receivers or optimize their effectiveness as a display [18]. If so, this may require some degree of structural representation in the signaller that causes them to signal more efficiently in large bouts. However, Menzerath’s Law has also been identified in ostensibly non-meaningful combinations of multiple call types (e.g. gelada contact calls [12]), which are unlikely to require any such representation. An alternative explanation is that the presence of multi-call-type sequences reflects a relatively high degree of communicative flexibility in a given behavioural context, which may extend to the duration of calls and intervals. However, it may also simply be reflective of a bias in the literature towards systems without uniform call sequences.

In conclusion, the call sequences produced by meerkats in submissive contexts represent an exception to the widespread [4] Menzerath’s Law at the level of vocal units but did adhere to it at the level of inter-call intervals. This finding highlights the nuanced ways in which compression can interact with vocal behaviour and the importance of examining systems in a multi-dimensional manner. An important further step would be to explore other contexts within the meerkat vocal repertoire [8]. One prediction would be that ecologically ‘urgent’ communicative contexts such as submission and predation events would have an acoustic structure with a highly optimized trade-off between perceptual saliency and production efficiency that does not lend itself to the kind of dynamic modifications necessary to support Menzerath’s Law. Less contextually urgent calls on the other hand, such as contact and feeding calls, may be more open to modulation of their temporal properties, rendering them subject to dynamic forces of compression such as Menzerath’s Law. By expanding this investigation to yet more species and behavioural contexts, it may eventually be possible to identify species-general regularities in where signals do, or do not, adhere to laws of compression.

## Data Availability

The data and R scripts used for our analysis and production of related figures can be freely accessed from our Open Science Framework repository: https://osf.io/vjgke/. Supplementary material is available online [54].
